# Metabonomics uncovers a reversible proatherogenic lipid profile during infliximab therapy of inflammatory bowel disease

**DOI:** 10.1186/s12916-017-0949-7

**Published:** 2017-10-16

**Authors:** Jacob Tveiten Bjerrum, Casper Steenholdt, Mark Ainsworth, Ole Haagen Nielsen, Michelle AC Reed, Karen Atkins, Ulrich Leonhard Günther, Fuhua Hao, Yulan Wang

**Affiliations:** 1 Department of Gastroenterology, Medical Section, Herlev Hospital, University of Copenhagen, Herlev Ringvej 75, DK-2730 Herlev, Denmark; 2 HWB-NMR, Institute of Cancer and Genomic Sciences, University of Birmingham, Edgbaston, Birmingham, UK; 3 Key Laboratory of Magnetic Resonance in Biological Systems, State Key Laboratory of Magnetic Resonance and Atomic and Molecular Physics, Wuhan Centre for Magnetic Resonance, Wuhan Institute of Physics and Mathematics, The Chinese Academy of Sciences, Wuhan, People’s Republic of China; 4 Collaborative Innovation Center for Diagnosis and Treatment of Infectious Diseases, Hangzhou, People’s Republic of China

**Keywords:** Crohn’s disease, Diagnostics, Metabolomics, Serum, Ulcerative colitis

## Abstract

**Background:**

One-third of inflammatory bowel disease (IBD) patients show no response to infliximab (IFX) induction therapy, and approximately half of patients responding become unresponsive over time. Thus, identification of potential treatment response biomarkers are of great clinical significance. This study employs spectroscopy-based metabolic profiling of serum from patients with IBD treated with IFX and healthy subjects (1) to substantiate the use of spectroscopy as a semi-invasive diagnostic tool, (2) to identify potential biomarkers of treatment response and (3) to characterise the metabolic changes during management of patients with tumour necrosis factor-α inhibitors.

**Methods:**

Successive serum samples collected during IFX induction treatment (weeks 0, 2, 6 and 14) from 87 IBD patients and 37 controls were analysed by ^1^H nuclear magnetic resonance (NMR) spectroscopy. Data were analysed with principal components analysis and orthogonal projection to latent structures discriminant analysis using SIMCA-P+ v12 and MATLAB.

**Results:**

Metabolic profiles were significantly different between active ulcerative colitis and controls, active Crohn’s disease and controls, and quiescent Crohn’s disease and controls. Metabolites holding differential power belonged primarily to lipids and phospholipids with proatherogenic characteristics and metabolites in the pyruvate metabolism, suggestive of an intense inflammation-driven energy demand. IBD patients not responding to IFX were identified as a potentially distinct group based on their metabolic profile, although no applicable response biomarkers could be singled out in the current setting.

**Conclusion:**

^1^H NMR spectroscopy of serum samples is a powerful semi-invasive diagnostic tool in flaring IBD. With its use, we provide unique insights into the metabolic changes taking place during induction treatment with IFX. Of distinct clinical relevance is the identification of a reversible proatherogenic lipid profile in IBD patients with active disease, which partially explains the increased risk of cardiovascular disease associated with IBD.

**Electronic supplementary material:**

The online version of this article (doi:10.1186/s12916-017-0949-7) contains supplementary material, which is available to authorized users.

## Background

Ulcerative colitis (UC) [1] and Crohn’s disease (CD) [2] are the two most prevailing entities of inflammatory bowel disease (IBD), characterised by chronic or recurrent episodes of intestinal inflammation. Both UC and CD present as multifactorial diseases believed to occur in genetically predisposed individuals due to an abnormal immunologic response to environmental and microbial components [3]. UC and CD share genetic and phenotypic characteristics; however, they are distinct diseases requiring precise differentiation to achieve optimal treatment regimens [4]. The diagnosis of IBD and the differentiation of UC and CD are based on a time-consuming and costly multidisciplinary approach (i.e. clinical history, endoscopy, radiology, histology, microbiology and haematology) that is hampered by the fact that approximately 10% of patients are left with the diagnosis of IBD unclassified [5]. Once a patient is diagnosed, selecting an individualised treatment regimen becomes the next challenging step, which is important because it involves costly biologic agents such as tumour necrosis factor-α (TNF-α) inhibitors, i.e. infliximab (IFX) [6, 7].

IFX is effective in inducing and maintaining remission of UC and luminal or fistulising CD. However, one-third of IBD patients show no response at all to IFX induction (i.e. primary non-responders) and up to half of patients responding to IFX become unresponsive over time (i.e. loss of response and thus withdrawal from treatment), whereas the remaining patients achieve long-term remission with continued IFX therapy [8–11]. Consequently, it is of great clinical and socioeconomic importance to identify biomarkers of efficacy of IFX before therapy is initiated. During ineffective IFX therapy, irremediable disease progression may take place, subsequently leading to severe outcomes. Further, the total cost of such unsuccessful treatments is an enormous burden on public health expenses.

In this respect, surprisingly few studies have focused on identifying predictive response biomarkers [12–14]. However, with the advent and feasibility of proton nuclear magnetic resonance (^1^H NMR) spectroscopy-based metabolic profiling [15, 16] of serum in IBD [17–21], metabonomics presents as an obvious tool for differential diagnoses and identification of predictive response biomarkers.

This study is a longitudinal cohort study of ^1^H NMR spectroscopy-based metabolic profiling of serum from IBD patients treated with IFX (1) to identify potential diagnostic biomarkers that hold differential power with respect to UC and CD patients and control subjects, (2) to provide insight into the disordered metabolism during active and quiescent IBD, and (3) to identify metabolic changes during treatment with IFX in order to explore markers of favourable outcomes.

## Methods

### Patient population

From May 2009 onward, blood samples were obtained from IBD patients prior to IFX infusion as part of a local drug monitoring program at the Department of Gastroenterology, Medical Section, Herlev Hospital, Denmark. All eligible UC (n = 49) and CD (n = 38) patients were naive to biologics, all had their diagnosis verified in accordance with well-established criteria [22, 23] and, at the time of IFX infusion, they were all graded in accordance with the Mayo score [24] (a score of 0–1: inactive UC, 2–4: mild UC, 5–8: moderate UC, and 9–12: severe UC), Harvey–Bradshaw (HB) score [25] (a score of 0–4: inactive CD, 5–8: mild CD, 9–16: moderate CD, and > 16: severe CD), and/or the perianal disease activity index [26] (PDAI; a score from 0 to 20, with a higher score indicating more severe disease). The healthy volunteer subjects (n = 37) were recruited locally among the staff at Herlev Hospital and were free of any daily medications (see Tables 1 and 2 for clinical details). Exclusion criteria were age above 80 or below 18 years; clinical evidence of any infections; recent (within 14 days) use of antibiotics, probiotics or high-dose prednisolone (≥60 mg/day); pregnancy; severe mental illness; and special food regimens such as a diet of fermentable oligosaccharides, disaccharides, monosaccharides and polyols, a diabetic diet or a gluten-free diet.

**Table 1:** Clinical details

	CD Total	CD Rem	CD Res	CD NRes	Control
Characteristics	n = 49	n = 29	n = 11	n = 9	n = 37
Gender (male/female)	22/27	15/14	4/7	3/6	18/19
Age, years (mean, range)	40 (19–71)	39 (22–62)	42 (20–63)	42 (19–58)	42 (26–63)
Age at diagnosis (≤25/> 25 years)	26/23	15/14	7/4	4/5	–
Years with disease (≤10/> 10 years)	27/22	16/13	6/5	5/4	–
HB-score (mean, range)	10 (6–18)^a^	10 (6–18)^c^	11 (8–15)	11 (5–15)^f^	–
PDAI (mean, range)	10 (2–12)^b^	9 (2–12)^d^	10 (9–12)^e^	7(6–9)^g^	–
Extension (D, J, TI, IC, C)	1, 1, 17, 5, 35	0, 0, 9, 3, 23	0, 0, 6, 1, 7	1, 1, 2, 1, 5	–
Surgery (IR, IR + HC, HC, Co, IC + Co)	9, 1, 2, 9, 4	5, 0, 2, 5, 0	2, 1, 0, 2, 2	2, 0, 0, 2,2	–
Smoking/non-smoking	13/36	11/18	1/10	1/8	0/37
EIM (present/not present)	4/45	1/28	2/9	1/8	–
Steroids, n					–
Independent/dependent/responder/unknown	7/20/8/14	4/12/3/10	2/4/2/3	1/4/3/1	–
Daily medication, n					–
Systemic 5-aminosalicylic acid (1.6–3.2 g)	2	1	0	1	–
Topical 5-aminosalicylic acid (1 g)	0	0	0	0	–
Systemic glucocorticoids (75 mg)^h^	9	6	2	1	–
Topical glucocorticoids (100 mg)	1	1	0	0	–
Azathioprine (100–150 mg)	29	18	7	4	–
Methotrexate (25 mg/wk)	1	0	0	1	–
None	15	9	4	2	37

^a^Six patients had no luminal activity, only perianal fistulas

^b^In all, fourteen patients had perianal fistulas

^c^Four patients had no luminal activity, only perianal fistulas

^d^In all, seven patients had perianal fistulas

^e^Three patients had perianal fistulas

^f^One patient had no luminal activity, only perianal fistulas

^g^In all, three patients had perianal fistulas

^h^Tappering regime

*C* colonic, *Co* colectomy, *D* duodenal, *EIM* extra-intestinal manifestations, *HB* Harvey–Bradshaw, *HC* hemicolectomy, *IC* ileocecal, *IR* ileocecal resection, *J* jejunal, *NRes* non-responder, *PDAI* perianal disease activity index, *Rem* remission, *Res* responder, *TI* terminal ileum

**Table 2:** Clinical details

	UC Total	UC Rem	UC Res	UC NRes	Control
Characteristics	n = 38	n = 19	n = 9	n = 10	n = 37
Gender (male/female)	18/20	9/10	4/5	5/5	18/19
Age, years (mean, range)	39 (20–66)	41 (20–66)	38 (23–65)	37 (21–54)	42 (26–63)
Age at diagnosis (≤25/> 25 years)	15/23	5/14	4/5	6/4	–
Years with disease (≤10/> 10 years)	24/14	10/9	6/3	8/2	–
Mayo-score (mean, range)	7 (3–12)	6 (3–12)	8 (6–11)	7 (5–10)	–
Extension (P, PS, LC, PC)	2/8/5/23	1/7/1/10	0/1/1/7	1/0/3/6	–
Surgery/no surgery	2/36	1/18	0/9	1/9	–
Smoking/non-smoking	2/36	1/18	1/8	0/10	0/37
EIM (present/not present)	0/38	0/19	0/9	0/10	–
Steroids, n					–
Independent/dependent/responder	1/32/5	1/17/1	0/8/1	0/7/3	–
Daily medication, n					–
Systemic 5-aminosalicylic acid (1.6–3.2 g)	29	13	7	9	–
Topical 5-aminosalicylic acid (1 g)	1	1	0	0	–
Systemic glucocorticoids (75 mg)^a^	18	9	4	5	–
Topical glucocorticoids (100 mg)	1	1	0	0	–
Azathioprine (100–150 mg)	25	14	5	5	–
None	0	0	0	0	37

^a^Tappering regime

*EIM* extra-intestinal manifestations, *LC* left-sided colitis, *NRes* non-responder, *P* proctitis, *PC* pancolitis, *PS* proctosigmoiditis, *Rem* remission, *Res* responder

### Classification of response to IFX

The outcome of IFX treatment was determined in accordance with previous studies [27, 28]:

1. Remission (Rem) was defined as a favourable clinical response to IFX induction (Mayo score < 2, HB score < 5, PDAI score < 5), followed by a sustained clinical remission at the initiation of maintenance therapy, i.e. at week 14.
2. Response (Res) was defined as a beneficial clinical response to IFX induction (reduced Mayo score, HB score and/or PDAI score) but without complete clinical remission (Mayo score ≥ 2, HB score ≥ 5, PDAI score ≥ 5) at initiation of maintenance therapy, i.e. at week 14 and despite subsequent dose optimisation.
3. Non-response (NRes) was defined as no clinical response to IFX induction therapy at weeks 2, 6 or 14.

### Serum sample collection and preparation

Blood samples were collected during the induction treatment with IFX and obtained as trough levels with sampling 30 min prior to an IFX infusion. Hence, samples were available from time-point 0 (before first infusion of IFX), 2 weeks after the initial dose (before the second infusion), 6 weeks after the initial dose (before the third infusion) and 14 weeks after the initial dose (before continuing maintenance therapy, i.e. the fourth infusion). Patients with severe disease and no initial response to IFX treatment often never received their third and fourth infusions of IFX. One sample was available from each control subject. In total, 359 samples were available for analysis from non-fasting patients and controls. Within 3 h of sampling, the serum was collected after centrifugation (2500 × *g* for 5 min at ambient temperature) and stored at −80 °C until analysis.

The serum was thawed and prepared for ^1^H NMR spectroscopy by mixing 180 μL of serum with 60 μL of 400 mM phosphate buffer in an Eppendorf tube to achieve a final concentration of 100 mM. A total of 180 μL from each of these mixed serum samples was transferred into 3-mm sample tubes, briefly centrifuged with a hand centrifuge to remove air bubbles, and subsequently placed in the high-throughput SampleJet robotic system used for the ^1^H NMR experiment.

### ^1^H NMR spectroscopy

All ^1^H NMR experiments were performed at 288.1 K using a Bruker 600-MHz spectrometer operating at 599.35 MHz for protons and equipped with an inverse detection 5-mm cryogenic probe (BioSpin, Bruker, Rheinstetten, Germany). For all samples, the ^1^H NMR spectra were acquired using a Carr–Purcell–Meiboom–Gill (CPMG) pulse sequence with an echo time of 160 ms. The 90-degree pulse length was approximately 8 μs, as calculated automatically for each sample. A total of 128 scans were collected into 64,000 data points with a spectral width of 14 ppm (8370 Hz; steady-state scans = 8; acquisition time = 3.9 s). Metabolites were assigned according to previously published data [29–31] and online databases for metabonomics research such as Human Metabolome Database and Madison Metabolomics Consortium Database.

### ^1^H NMR spectroscopy procession

The free-induction decays for one-dimensional data were zero-filled to 64,000 data points and multiplied by an exponential function with a line-broadening factor of 1 Hz prior to Fourier transformation. All one-dimensional ^1^H NMR spectra were manually corrected for phase using TOPSPIN 3.1 (BioSpin, Bruker) and corrected for baseline distortions, and referenced to the left signal peak of α-glucose at 5.236 ppm using Mnova 8.1 (Mestrelab Research, Santiago de Compostela, Spain). The ^1^H NMR spectral region 0.5–9.0 ppm was binned with a width of 0.004 ppm (2 Hz) using the Mnova software. Water regions (4.23–5.18 ppm) were removed to avoid imperfect water saturation. The regions 1.43–1.50, 6.98–7.10, and 7.70–7.84 ppm, were aligned to avoid the effect of chemical-shift drift. The ^1^H NMR spectra were normalised to the total sum of the spectral integrals to compensate for sample concentration differences.

### Multivariate data analysis

The applied approach to multivariate data analysis has previously been described in detail by Bjerrum et al. [32].

To visualise the general structure of each ^1^H NMR dataset and to identify any abnormalities or outliers (based on the principles of Hotelling’s *T* ^2^) within the data, principal components analyses were completed on mean-centred data (SIMCA-P+ software v12.0, Umetrics, Umeå, Sweden). Projection to latent structures discriminant analysis (PLS-DA) and orthogonal projection to latent structures discriminant analysis (O-PLS-DA) [33] were subsequently applied to the ^1^H NMR spectral data scaled to unit variance to discover metabolic differences. O-PLS is an extension of the partial least squares regression method, including an integrated orthogonal signal-correction filter [34]. As described by Cloarec et al. [35], analysis of the model is facilitated by a back-scaled transformation of the loadings, with incorporated colour-coded correlation coefficients (MATLAB v7.1, MathWorks, Natick, MA, USA) of the metabolites accountable for the differentiation. Briefly, each back-scaled loading is plotted as a function of the respective chemical shift with a colour code that signifies the weights of the discriminatory variables. A hot colour (e.g. red) signifies metabolites that are markedly different between groups, whereas a cool colour (e.g. blue) corresponds to no differences between groups.

To minimise the risk of overfitting and to validate the PLS models, a sevenfold cross-validation procedure was performed, involving iterative construction of models by repeatedly leaving out one-seventh of the samples and predicting them back into the model. This resulted in a cross-validation parameter, *Q* ^2^, indicating the predictability of the model in relation to its statistical validity. Furthermore, a cross-validation procedure, a permutation test, was completed for each model, wherein 200 models were constructed with the use of randomised classification of the samples, and *Q* ^2^ values were generated from these models and compared with the *Q* ^2^ values of the real model. If the maximum value of ${\mathsf{Q}}_{\mathsf{max}}^2$ from the permutation test was smaller than the *Q* ^2^ of the real model, the model was regarded as a predictable model. Similarly, *R* ^2^ was used to evaluate possibly overfitted models. Finally, with the use of an analysis of variance of the cross-validated residuals (CV-ANOVA) [36] test, a significance test was implemented to verify model validity. Only if the permutation test and the CV-ANOVA test were fulfilled at the same time were the models considered valid. The actual predictive value of each model was estimated with the use of receiver operating characteristics.

Correlation analyses between metabolites were performed with the use of the respective resonance peaks from the NMR spectra.

## Results

### Classification of clinical outcome

**Fig. 1:**
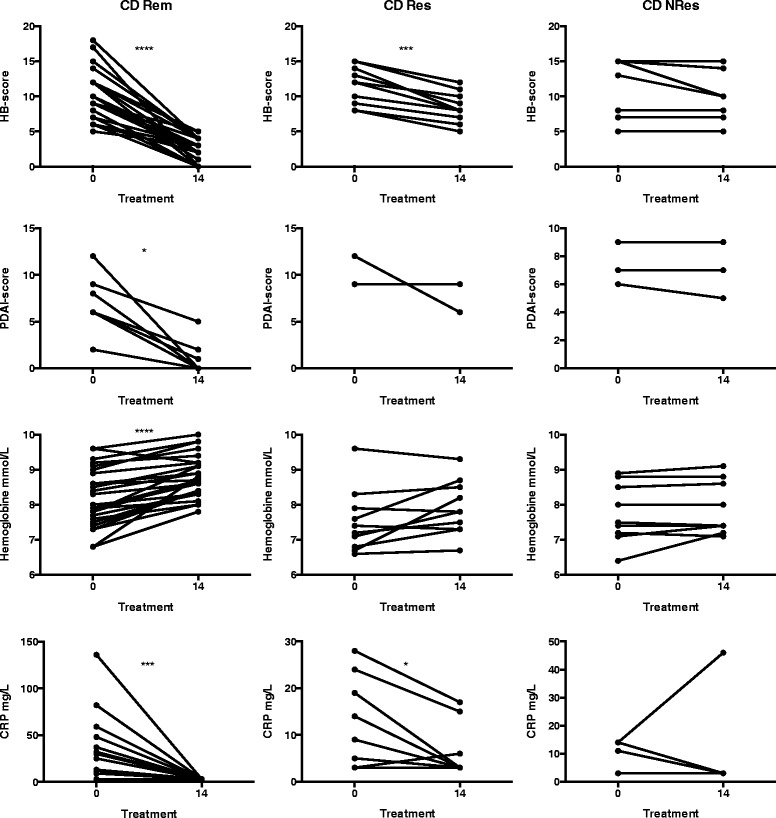
Markers of disease activity in patients with Crohn’s disease (CD). Changes in Harvey–Bradshaw (HB) scores, perianal disease activity index (PDAI) and biochemical markers (C-reactive protein and haemoglobin levels) of disease activity during treatment of CD patients with infliximab (IFX) measured at the time of IFX initiation (0) and at the fourth infusion (14). *Rem* remission, *Res* responder, *NRes* non-responder. * *P* < 0.05; *** *P* < 0.001; **** *P* < 0.0001

**Fig. 2:**
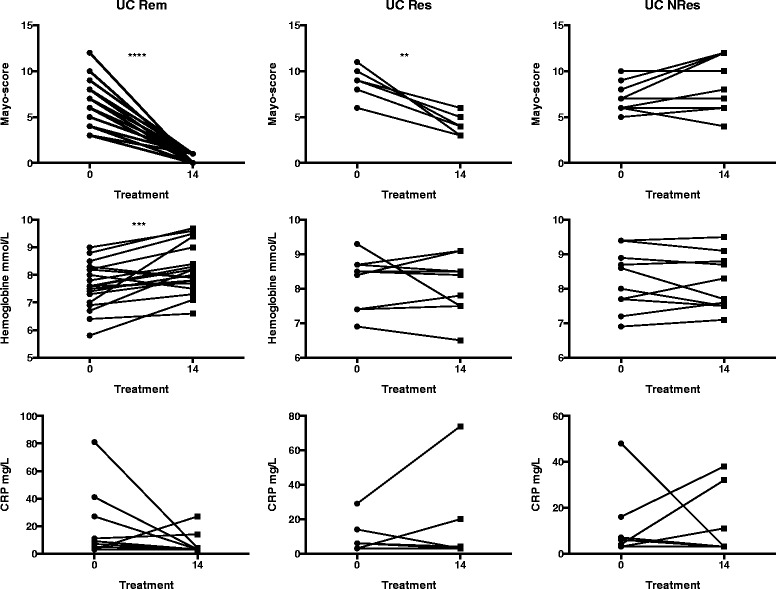
Markers of disease activity in patients with ulcerative colitis (UC). Changes in Mayo scores and biochemical markers (C-reactive protein and haemoglobin levels) of disease activity during treatment of UC patients with infliximab (IFX) measured at the time of IFX initiation (0) and at the fourth infusion (14). *Rem* remission, *Res* responder, *NRes* non-responder. ** *P* < 0.01; *** *P* < 0.001; **** *P* < 0.0001

Patients with UC and CD were primarily classified into three major groups stated above –Rem, Res and NRes. These classifications were based on clinical scores (Mayo or HB score and PDAI score) and concomitant biochemical parameters (Figs. 1 and 2). Patients with CD who went into remission had significant decreases in HB and/or PDAI scores and C-reactive protein (CRP), and an increase in haemoglobin. CD responders also had significant decreases in HB score and CRP, but no significant changes were seen in PDAI score or haemoglobin. CD non-responders experienced no significant changes at all (Fig. 1). Similarly, UC patients who went into remission had a significant decrease in Mayo score and an increase in haemoglobin and a trend toward decreased CRP. UC responders had a significant decline in Mayo score but without significant changes in haemoglobin or CRP. UC non-responders had no changes at all (Fig. 2).

### ^1^H NMR spectra of serum

Typical ^1^H NMR spectra of serum obtained from patients with CD and UC as well as control subjects are shown in Additional file 1: Figure S1. A range of endogenous metabolites observed in the spectra is similar to that of previously reported metabolites [17–21] and consisted of different amino acids (e.g. valine, leucine, isoleucine, alanine, arginine, lysine, glutamine, glutamate, histidine, glycine, tyrosine and phenylalanine), short-chain fatty acids (e.g. acetate and isobutyrate), energy metabolism-related molecules (e.g. lactate, citrate and creatine), and membrane metabolites, including glycerophosphocholine (GPC) and choline. Notably, a range of lipids and lipoproteins (e.g. high-density lipoproteins (HDLs), low-density lipoproteins (LDLs) and very low-density lipoproteins (VLDL)) was also detected in the NMR spectra.

A number of unknown metabolites (U1, U2 and U3) was identified, but further characterisation was impossible in spite of correlation analysis with STOCSY (statistical total correlation spectroscopy) and database search.

### Multivariate data analysis of ^1^H NMR spectra

#### Unsupervised analysis and outlier detection

Principal components analyses was initially applied to the NMR spectra acquired from the serum using data scaled to unit variance. Based on the principals of Hotelling’s *T* ^2^ (95% confidence limit), 16 samples were identified as outliers and subsequently excluded. The outliers were reviewed for any demographic abnormalities, but none could be identified. Thus, the remaining 343 samples were accessible for subsequent supervised multivariate data analysis (Additional file 2: Table S1).

#### Supervised analysis of phenotypes and identification of metabolites

To classify the samples, PLS-DA models comparing control subjects with either UC or CD patients, UC versus CD, treatment response types (i.e. Rem, Res and Nres), and different disease states (i.e. weeks 0, 2, 6 and 14) during the 14 weeks of treatment with IFX were generated, with CPMG NMR data as an **X** matrix and class information as the *Y* variables [35]. To uncover the metabolic changes holding differential power, the O-PLS-DA strategy was subsequently applied to each model. The O-PLS-DA models were constructed with one PLS component and one orthogonal component. The models were subsequently validated with the use of a permutation test (for the PLS-DA models) and a CV-ANOVA test (for the O-PLS-DA models).

**Fig. 3:**
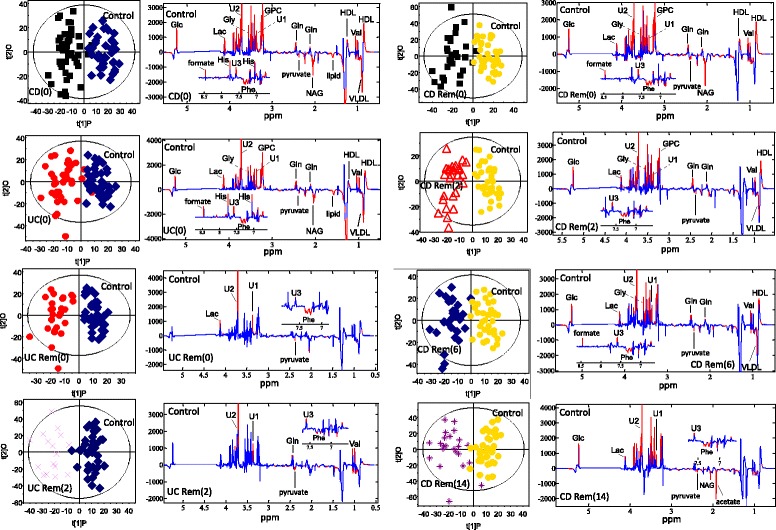
O-PLS-DA score plots and loading plots. O-PLS-DA score plots and corresponding coefficient-coded loading plots obtained from metabolic profiles of ^1^H NMR spectra of the serum samples. The score plots display the first PLS component and one orthogonal component for each model. A two-way separation of the samples is demonstrated in all plots. The corresponding back-scaled loading plots reflect the class differences in the NMR spectra. Upright peaks indicate a relatively increased intensity of metabolites, and downright peaks a decreased intensity of metabolites. The colours shown on the plot are associated with the significance of metabolites in separating the samples; red indicating significance at a level of *P* < 0.05. (0), before first infusion of infliximab; (2), before second infusion; (6), before third infusion; (14), before fourth infusion. *Glc* glucose, *Gln* glutamine, *GPC* glycerophosphocholine, *Gly* glycine, *HDL* high-density lipoproteins, *His* histidine, *Lac* lactate, *NAG* N-acetyl glycoprotein, *Phe* phenylalanine, *U1*, *U2*, *U3* unknown metabolite, *Val* valine, *VLDL* very-low density lipoproteins, *CD* Crohn’s disease, *UC* ulcerative colitis, *Rem* remission

As seen in Table 3, no significant models could be created for CD(0) versus UC(0), indicating that no differences in the metabolic profiles of active CD and UC could be identified. Both CD(0) and UC(0), however, were easily distinguished from control subjects, which was also true for CD Rem at all time points during IFX treatment (Table 3). In contrast, UC Rem became inseparable from control subjects during the 14 weeks of IFX treatment as patients went into remission (Table 3). The corresponding score plots and their back-scaled loading plots of the significant models are shown in Fig. 3, where a clear separation between each class is seen in the score plots. The corresponding back-scaled loading plots reflect the class differences of the NMR spectra and indicate relatively increased or decreased intensities of metabolites. These back-scaled loading plots were converted into a comprehensive list of markedly up- and down-regulated metabolites in Table 4. The actual prediction performance estimates are presented in Table 3 as area under the curve (AUC) and correlate with the results of the OPLS-DA models; all significant models produce AUC values above 90. The AUCs are presented in Additional file 3: Figure S2.

**Table 3:** Validation of PLS-DA and O-PLS-DA models

Model	PLS-DA permutation test n = 200	O-PLS-DA CV-ANOVA	Area under the ROC curve
CD(0) vs. UC(0)	Q^2^ = 0.095	Q^2^ = –0.182	0.44
	×	× *P* = 1	
CD(0) vs. Control	Q^2^ = 0.637, r = 0.32	Q^2^ = 0.7	0.96
	✓	✓*P* < 0.001	
UC(0) vs. Control	Q^2^ = 0.583, r = 0.32	Q^2^ = 0.383	0.94
	✓	✓*P* < 0.001	
CD Rem(0) vs. Control	Q^2^ = 0.63, r = 0.37	Q^2^ = 0.66	0.96
	✓	✓*P* < 0.001	
CD Rem(2) vs. Control	Q^2^ = 0.69, r = 0.37	Q^2^ = 0.66	0.95
	✓	✓*P* < 0.001	
CD Rem(6) vs. Control	Q^2^ = 0.52, r = 0.37	Q^2^ = 0.63	0.91
	✓	✓*P* < 0.001	
CD Rem(14) vs. Control	Q^2^ = 0.48, r = 0.40	Q^2^ = 0.62	0.90
	✓	✓*P* < 0.001	
UC Rem(0) vs. Control	Q^2^ = 0.56, r = 0.47	Q^2^ = 0.60	0.91
	✓	✓*P* < 0.001	
UC Rem(2) vs. Control	Q^2^ = 0.60, r = 0.48	Q^2^ = 0.60	0.94
	✓	✓*P* < 0.001	
UC Rem(6) vs. Control	Q^2^ = 0.25	Q^2^ = 0.52	0.71
	×	✓*P* < 0.001	
UC Rem(14) vs. Control	Q^2^ = 0.17	Q^2^ = 0.37	0.64
	×	✓*P* < 0.001	

The models were only considered valid if the permutation test and the CV-ANOVA test (p < 0.05) were satisfied at the same time

(0), before 1st infusion of infliximab; (2), before 2nd infusion; (6), before 3rd infusion; (14), before 4th infusion

Q2, predictability of the model; r correlation coefficient

✓, valid model

X invalid model

*CD* Crohn’s disease, *CV*-*ANOVA* analysis of variance of the cross-validated residuals, *O*-*PLS*-*DA* orthogonal-projection to latent structure discriminant analysis, *PLS*-*DA* projection to latent structure-discriminant analysis, *Rem* remission, *ROC* receiver operating characteristics, *UC* ulcerative colitis

**Table 4:**
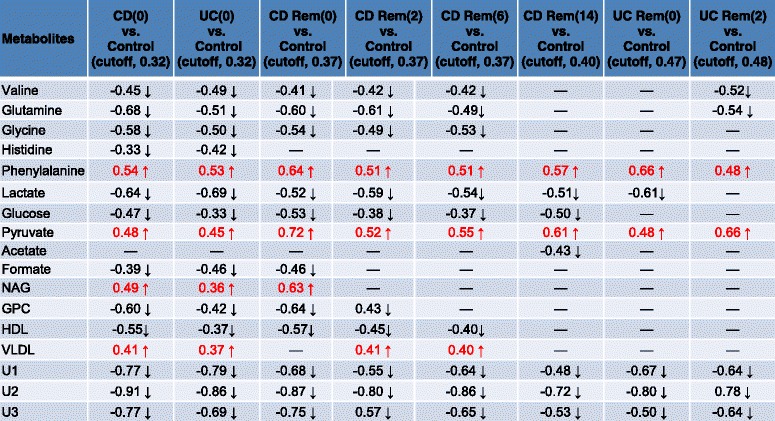
Significant up and down-regulated metabolites

(0), before 1st infusion of infliximab at week 0; (2), before 2nd infusion at week 2; (6), before 3rd infusion at week 6; (14), before 4th infusion at week 14

increased or ↓ decreased compared to controls (The coefficients from the OPLS-DA results, positive and negative signs, indicate positive and negative correlation in the concentrations, respectively. The coefficient of 0.32–0.48 was used as the cut-off value in the different models (according to number of samples) for the evaluation of significant differences (*P* < 0.05)

*CD* Crohn’s disease, *GPC* glycerophosphocholine, *HDL* high-density lipoprotein, *NAG* N-acetyl glycoprotein, *Rem* remission, *U1*, *U2*, *U3* unknown metabolites, *UC* ulcerative colitis, *VLDL* very-low density lipoprotein

Similar assessments were made between Res patients and control subjects, between NRes patients and control subjects, and between the different response types (i.e. Rem, Res and NRes patients; Additional file 4: Table S2). CD Res patients and UC Res patients could be distinguished from control subjects throughout the IFX induction period, whereas CD NRes patients and UC NRes patients could not. Furthermore, the comparisons between the different response types resulted in insignificant models.

Next, the potential metabolic changes that take place during the 14 weeks of IFX treatment were investigated by comparing the consecutive time points within each response type (i.e. Rem, Res and NRes in UC and CD patients separately). Surprisingly, no significant models could be identified (Additional file 5: Table S3; Additional file 6: Table S4).

Finally, a correlation analysis was performed between phenylalanine and tyrosine as phenylalanine is related to the tyrosine metabolism and was found up-regulated in IBD. This resulted in a significant positive correlation (r = 0.447, *P* < 0.001, Additional file 7: Figure S3).

#### Supervised analysis of subphenotypes

A final analysis was performed on the different subphenotypes where statistically possible; these were sex, age at diagnosis (≤30, 31–40, ≥ 41 years), years with diagnosis (≤10 years/> 10 years), Mayo and HB scores (mild, moderate and severe), fistulising disease (no/yes), stenosis (no/yes), extent of inflammation (left-sided colitis/pancolitis), surgery (no/yes), smoking status (no/yes), extraintestinal manifestations (present/not present) and glucocorticoids (dependent/independent/responder). This was completed by creating PLS-DA models for each patient category, with NMR data as an **X** matrix and phenotype information as the *Y* variables. None of the models, however, turned out to be predictive (Additional file 5: Table S3 and Additional file 6: Table S4).

## Discussion

This ^1^H NMR spectroscopy-based metabonomics study of serum samples demonstrates the differential power and metabolic differences present between IBD patients and control subjects and the changes taking place during 14 weeks of IFX treatment in different treatment response types (i.e. Rem, Res or primary NRes).

### Differentiation of CD and UC patients, control subjects and IFX response types

The study confirms previously published studies [17–21] and displays significant differential diagnostic power in models comparing active UC patients versus control subjects, active CD patients versus control subjects, and remission CD patients versus control subjects but no significant discrimination for active UC versus active CD patients, active UC versus remission UC patients, active CD versus remission CD patients, and remission UC patients versus control subjects (Table 3 and Additional file 5: Table S3 and Additional file 6: Table S4). No previous studies have actually compared the active and quiescent disease stages of CD and UC patients, although Dawiskiba et al. [17] did compare active IBD and remission IBD patients and found significant metabolic profiles that differentiated these two congregated cohorts. They also compared remission IBD patients and control subjects, but without any significant results. This, however, contrasts with the findings of Williams et al. [20], who were able to differentiate between remission CD patients and control subjects, as in the present study, and remission UC patients and control subjects, in contrast to the present study. The methodology and statistical procedures in the study by Williams et al. [20] and those used herein are comparable, and no obvious explanation has been identified for the discrepancy in outcome.

The sustained metabolic changes at 14 weeks of IFX treatment in the remission CD patients versus control subjects might be an indication of the more profound inflammation compared with the relatively superficial inflammation seen in UC patients; in Table 4, the number of significant up- or down-regulated metabolites decreases as treatment proceeds from the initial IFX infusion (CD Rem(0) vs. control) to the fourth infusion (CD Rem(14) vs. control). In contrast, patients with UC have fewer significant metabolic changes at the time of treatment initiation (UC Rem(0) vs. control), and these changes are already absent after 6 weeks of treatment. This circumstance might also explain why the metabolic profiles from the successive serum samples (i.e. weeks 0, 2, 6 and 14) within each response type (i.e. Rem, Res and NRes) did not show any significant discrimination (Additional file 5: Table S3 and Additional file 6: Table S4); the metabolic changes seen from week 0 through week 14 in patients with CD entering remission are simply too few (Table 4). Future studies need to collect serum samples within an expanded time frame, at least in CD patients. In contrast, patients with UC entering remission have very few significantly deregulated metabolites at the time of treatment initiation (Table 4, UC Rem(0) vs. control), and the subsequent metabolic changes may consequently be insufficient to create significant discrimination between successive serum samples.

As could be expected, the metabolic profiles of CD Res and UC Res patients stayed significantly different from that of control subjects (Additional file 4: Table S2) throughout the induction period. Surprisingly, this was not the case for CD NRes and UC NRes patients. The obvious explanation is the relatively low number of samples in these groups (n = 9 and 10, respectively), but it might also be explained by an entirely different metabolic profile in the serum of patients who are primary non-responders to IFX. However, comparing the different response types (i.e. Rem, Res and NRes) did not produce any significant discrimination. Thus, in order to answer this question, lager cohorts are needed in future studies. Furthermore, the lack of discriminant power might also be explained by the poor correlation in general between clinical disease activity indices and the actual burden of disease, although we did incorporate biochemical parameters to substantiate the clinical scores.

### Metabolic profiles of IFX-treated IBD patients

An abnormal lipid metabolism was identified in both CD and UC patients compared with control subjects. LDL data could not be separated from the broad lipoprotein NMR peak, but the identified low levels of HDL and high levels of VLDL seem to be the consequence of malabsorption, increased transit times and the inflammatory environment (e.g. TNF-α, interleukin-1 and -6). TNF-α in particular has been shown to increase the release of free fatty acids from adipocytes, stimulate the production of triglycerides in the liver, and inhibit lipoprotein lipase and thereby the hydrolysis of triglycerides in VLDL, leading to an increase in VLDL and a decrease in HDL cholesterols [37]. This proatherogenic lipid profile correlates with the increased incidence of cardiovascular morbidity seen in IBD patients, particularly in women and adolescents [38]. As seen in this study (Table 4) and others [39, 40], treatment with IFX ameliorates this proatherogenic lipid profile in both UC and CD patients. Thus, women and young adults with chronically active disease or frequent flares might be considered candidates for concomitant statin treatment if other atherogenic risk factors are present. However, the biobank from which the samples originated did not contain patient data on quantitative lipid measurements or other potential atherogenic risk factors except for sex, age and smoking.

This study also demonstrated decreased levels of the membrane metabolite GPC (Table 4). GPC is a derivative of the membrane metabolite choline and one of the major forms of choline storage in the cytosol. The decreased levels of GPC may be a consequence of the compromised integrity of the intestinal mucosal membrane [15] with an inflammation-driven increased rate of apoptosis and cellular turnover [41]. Interestingly, sustained choline deprivation stimulates cell survival through nuclear factor-kappa B activation, leading to sustained inflammation and oncogenesis [42]. Furthermore, low levels of GPCs have been correlated with cardiovascular disease [43], underlining the proatherogenic nature of the inflammation.

In contrast to other studies [17, 19–21], low levels of glucose and lactate were found in both UC and CD patients compared with control subjects, and these changes persisted in CD patients achieving remission after 14 weeks of IFX treatment (Table 4). The low level of glucose could be explained by impaired intestinal absorption due to the inflammation, which is more profound in CD than UC. However, the concomitant low level of lactate and high level of pyruvate indicate the need for scavengers of reactive oxygen species [44] and an inflammation-driven high energy demand with high glycolytic activity and low lactate dehydrogenase activity and hence accumulation of pyruvate but no production of lactate [45].

Phenylalanine was persistently up-regulated and positively correlated with tyrosine in IBD patients even during induction of remission, especially in patients with CD (Table 4). This is in contrast to the other observed amino acids (i.e. valine, glutamine, glycine and histidine; Table 4). However, the pattern of increased aromatic amino acids (e.g. phenylalanine and tyrosine) and a decrease in branched chain amino acids (e.g. valine) is well known during catabolic conditions like sepsis and liver failure [46, 47]. The inflammation-driven process causes muscle proteolysis with a subsequent decrease in branched chain amino acids due to muscle energy consumption and an increase in aromatic amino acids due to hepatic insufficiency with impairment of the hepatic enzyme phenylalanine-4-hydroxylase [48].

Formate is an essential intermediary metabolite in virtually all living organisms, but in this study, formate was found in low levels in IBD patients (Table 4). This potentially reflects the high demand of formate locally in the inflamed intestines, where formate is tightly related to folate metabolism [49]. A previous study [15] has indicated an increased phospholipid metabolism (e.g. GPC and choline) and glutathione synthesis (e.g. glutamine and glycine) in inflamed colonic tissue from patients with UC, and both of these processes are linked via the methionine-homocysteine cycle, where formate and folate are essential and central elements [49].

*N*-Acetyl glycoprotein is a well-known acute-phase protein, which is in accordance with the high levels seen in this study (Table 4), but due to a long half-life, it is not applicable as a useful clinical tool [50]. Finally, three unidentified metabolites were consistently down-regulated in both active and inactive patients with IBD (Table 4).

## Conclusions

^1^H NMR spectroscopy-based metabonomics on successive serum samples from patients with IBD has proven to be a potentially powerful semi-invasive diagnostic tool in flaring UC and CD, providing unique insights into the metabolic changes taking place during induction treatment with IFX. Thus, the identification of a reversible proatherogenic lipid profile in IBD patients with active disease might be of clinical importance in terms of statin treatment of IBD patients with atherogenic risk factors and continuous inflammation or frequent flares. Unfortunately, no discriminant biomarkers could be associated with the differentiation of CD and UC, nor could actual response biomarkers be identified, although IBD patients not responding to IFX were characterised as a potentially distinct group. However, with the increasing number of biological treatment options (e.g. TNF-α inhibitors and integrin antagonists) [51] and novel small molecules (e.g. Janus kinase inhibitors) [52], it will become imperative to develop predictive response biomarkers in order establish relational individualised medical treatment regimes.

## Additional files

Additional file 1: Figure S1. ^1^H NMR spectra of serum and metabolite assignment. (DOCX 116 kb)

Additional file 2: Table S1. Number of subjects and serum samples. (DOCX 18 kb)

Additional file 3: Figure S2. Area under the receiver operating characteristic (ROC) curve. (DOCX 211 kb)

Additional file 4: Table S2. Validation of PLS-DA and O-PLS-DA models. (DOCX 24 kb)

Additional file 5: Tables S3. Validation of PLS-DA and O-PLS-DA models of UC phenotypes and treatment response. (DOCX 23 kb)

Additional file 6: Tables S4. Validation of PLS-DA and O-PLS-DA models of CD phenotypes and treatment response. (DOCX 24 kb)

Additional file 7: Figure S3. Correlation analysis between phenylalanine and tyrosine. (DOCX 49 kb)

## Abbreviations

AUC: area under the curve
CD: Crohn’s disease
CPMG: Carr–Purcell–Meiboom–Gill
CRP: C-reactive protein
CV-ANOVA: analysis of variance of the cross-validated residuals
GPC: glycerophosphocholine
HB: Harvey–Bradshaw
HDL: high-density lipoproteins
IBD: inflammatory bowel disease
IFX: infliximab
LDL: low-density lipoproteins
NMR: nuclear magnetic resonance
NRes: non-responder
O-PLS-DA: orthogonal projection to latent structures discriminant analysis
PDAI: perianal disease activity index
PLS-DA: projection to latent structures discriminant analysis
Rem: remission
Res: response
TNF-α: tumour necrosis factor-α
UC: ulcerative colitis
VLDL: very low-density lipoproteins
