# Supplementary material for: Metabonomics uncovers a reversible proatherogenic lipid profile during infliximab therapy of inflammatory bowel disease
Source: BMC Med. 2017 Oct 16;15:184. doi: 10.1186/s12916-017-0949-7 (PMC5641999; doi:10.1186/s12916-017-0949-7)
Supplement: Supplementary file 1 — 1H NMR spectra of serum and metabolite assignment. (DOCX 116 kb) [file 12916_2017_949_MOESM1_ESM.docx]

**Additional file 1: Figure S1 ^1^H NMR spectra of serum and metabolite assignment.**


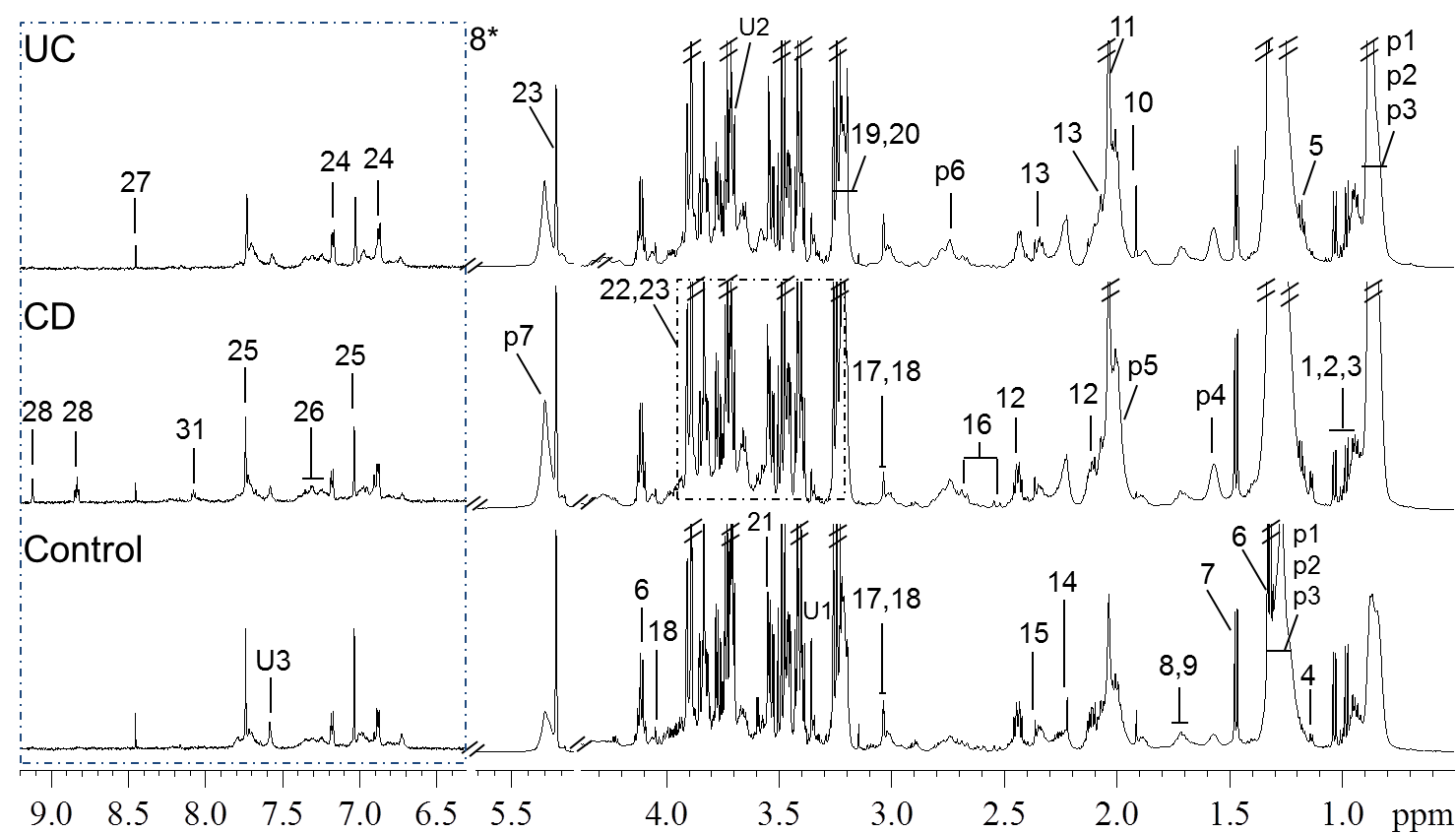


CD, Crohn’s disease; UC, ulcerative colitis

1, valine; 2, leucine; 3, isoleucine; 4, isobutyrate; 5,ethanol; 6, lactate; 7, alanine; 8, lysine; 9, arginine; 10, acetate; 11, N-acetyl glycoprotein (NAG); 12, glutamine; 13, glutamate; 14, acetoacetate; 15, pyruvate; 16, citrate; 17, creatine; 18, creatinine; 19, choline; 20, glycerophosphocholine (GPC); 21, glycine; 22, b-glucose; 23, α-glucose; 24, tyrosine; 25, histidine; 26, phenylalanine; 27, formate; 28, Trigonelline; U1, U2, U3, unknown metabolites; p1, high density lipoproteins (HDL); p2, low density lipoproteins (LDL); p3, very low density lipoproteins (VLDL); p4, lipids; p5, lipid (unsatured fatty acids); p6, polyunsatured fatty acids (PUFA); p7, unsatured fatty acids (UFA)
