# Supplementary material for: Metabonomics uncovers a reversible proatherogenic lipid profile during infliximab therapy of inflammatory bowel disease
Source: BMC Med. 2017 Oct 16;15:184. doi: 10.1186/s12916-017-0949-7 (PMC5641999; doi:10.1186/s12916-017-0949-7)
Supplement: Supplementary file 2 — Number of subjects and serum samples. (DOCX 18 kb) [file 12916_2017_949_MOESM2_ESM.docx]

| **Additional file 2: Table S1 Number of subjects and serum samples** | CD Total | CD Rem | CD Res | CD NRes | Control |
| --- | --- | --- | --- | --- | --- |
| Number of subjects | n = 49 | n = 29 | n = 11 | n = 9 | n = 37 |
| Number of samples at  0, 2, 6 and 14 weeks | 46,48,46,34 | 29,29,28,24 | 8,11,11,8 | 9,9,7,2 | 37 |
|  |  |  |  |  |  |
|  | UC Total | UC Rem | UC Res | UC NRes | Control |
| Number of subjects | n = 38 | n = 19 | n = 9 | n = 10 | n = 37 |
| Number of samples at  0, 2, 6 and 14 weeks | 35,36,31,30 | 17,17,19,18 | 9,9,7,7 | 9,10,5,5 | 37 |

CD, Crohn’s disease; NRes, non-responder; Rem, remission; Res, responder; UC, ulcerative colitis
