# Supplementary material for: Metabonomics uncovers a reversible proatherogenic lipid profile during infliximab therapy of inflammatory bowel disease
Source: BMC Med. 2017 Oct 16;15:184. doi: 10.1186/s12916-017-0949-7 (PMC5641999; doi:10.1186/s12916-017-0949-7)
Supplement: Supplementary file 3 — Area under the receiver operating characteristic (ROC) curve. (DOCX 211 kb) [file 12916_2017_949_MOESM3_ESM.docx]

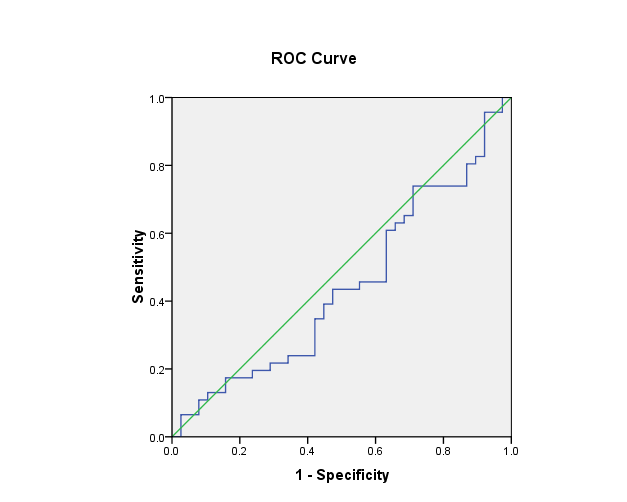

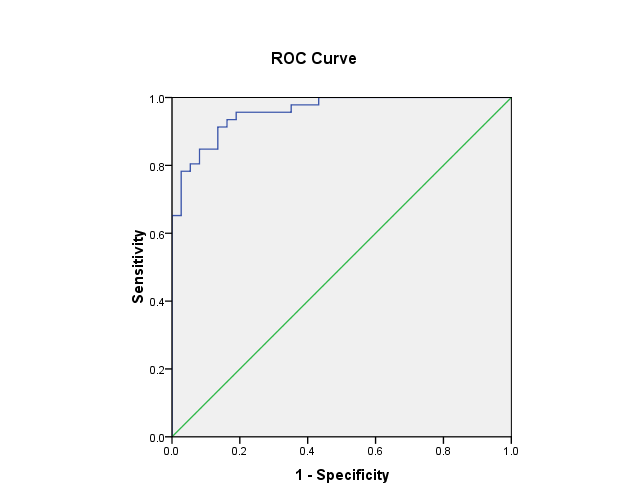

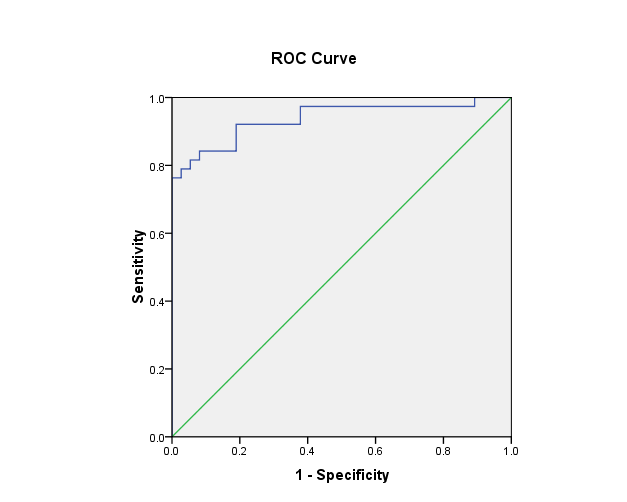

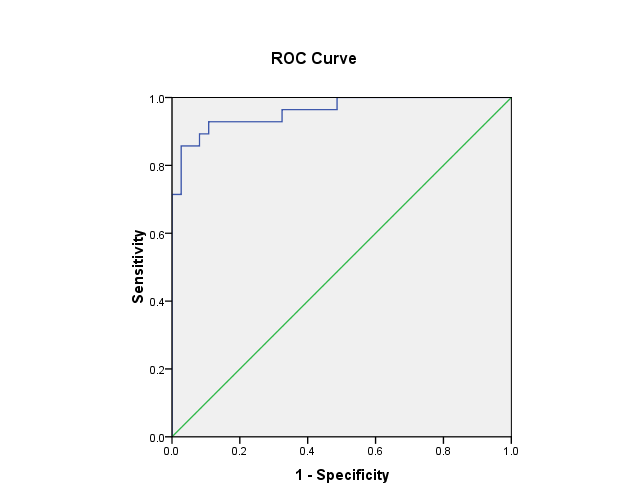

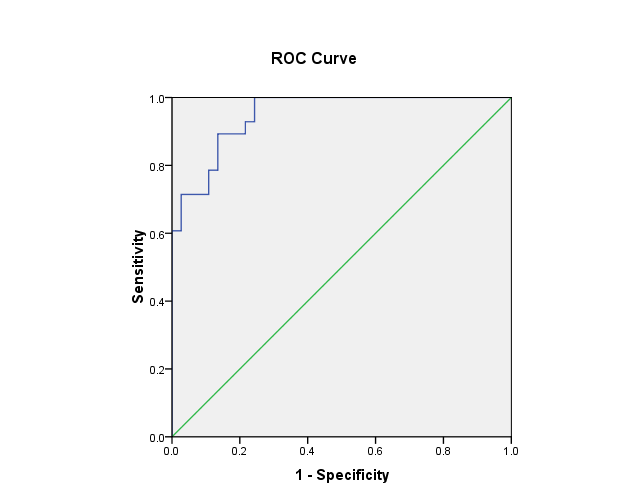

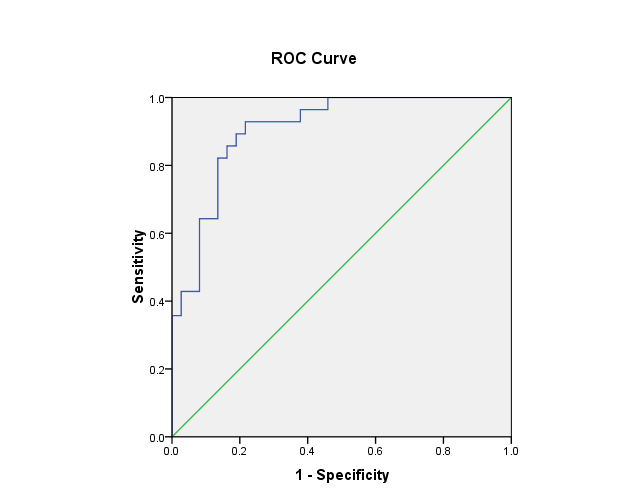

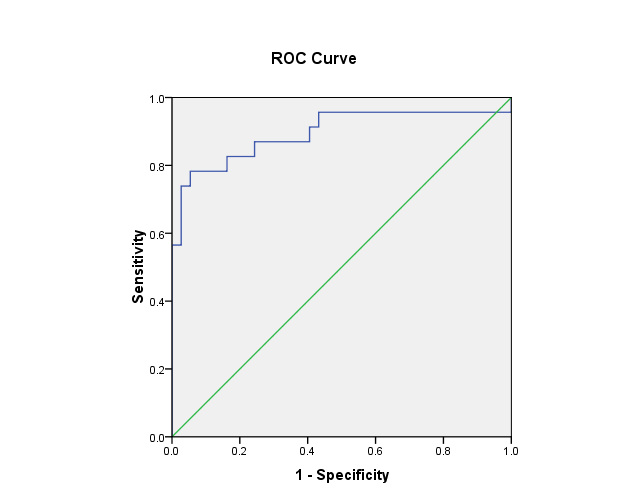

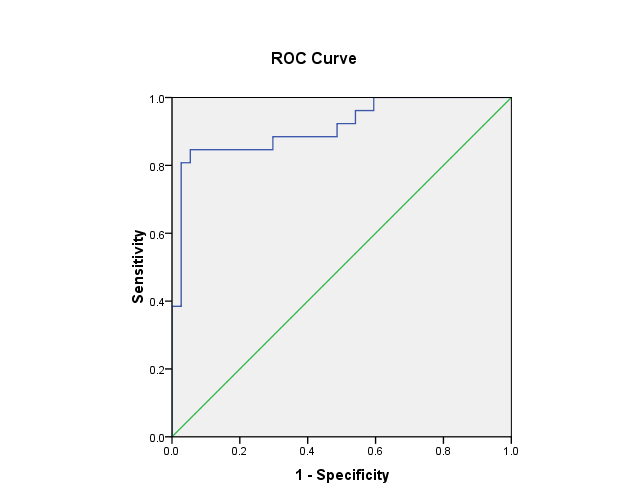

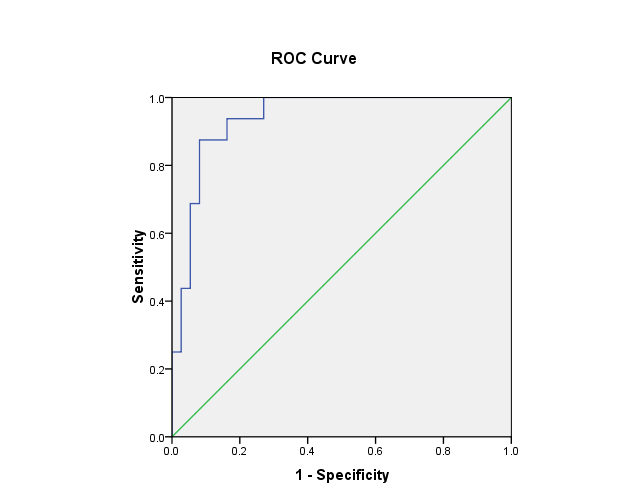

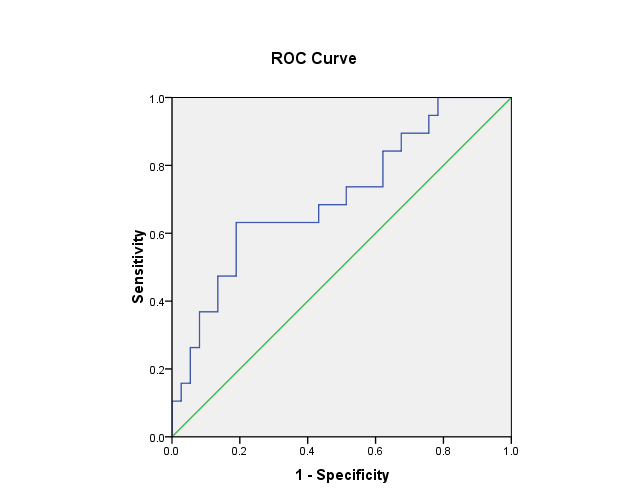

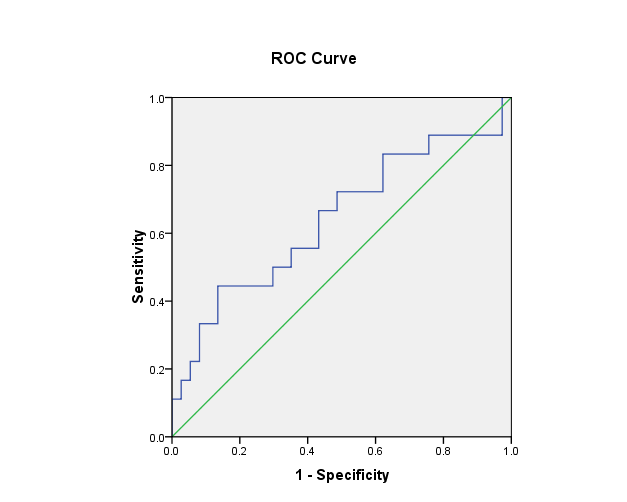


CD(0) vs. UC(0)

AUC, area under the receiver operating characteristic curve; CD, Crohn’s disease; Rem, remission; UC, ulcerative colitis

(0), before 1^st^ infusion of infliximab; (2), before 2^nd^ infusion; (6), before 3^rd^ infusion; (14), before 4^th^ infusion

AUC 0.64

AUC 0.72

AUC 0.94
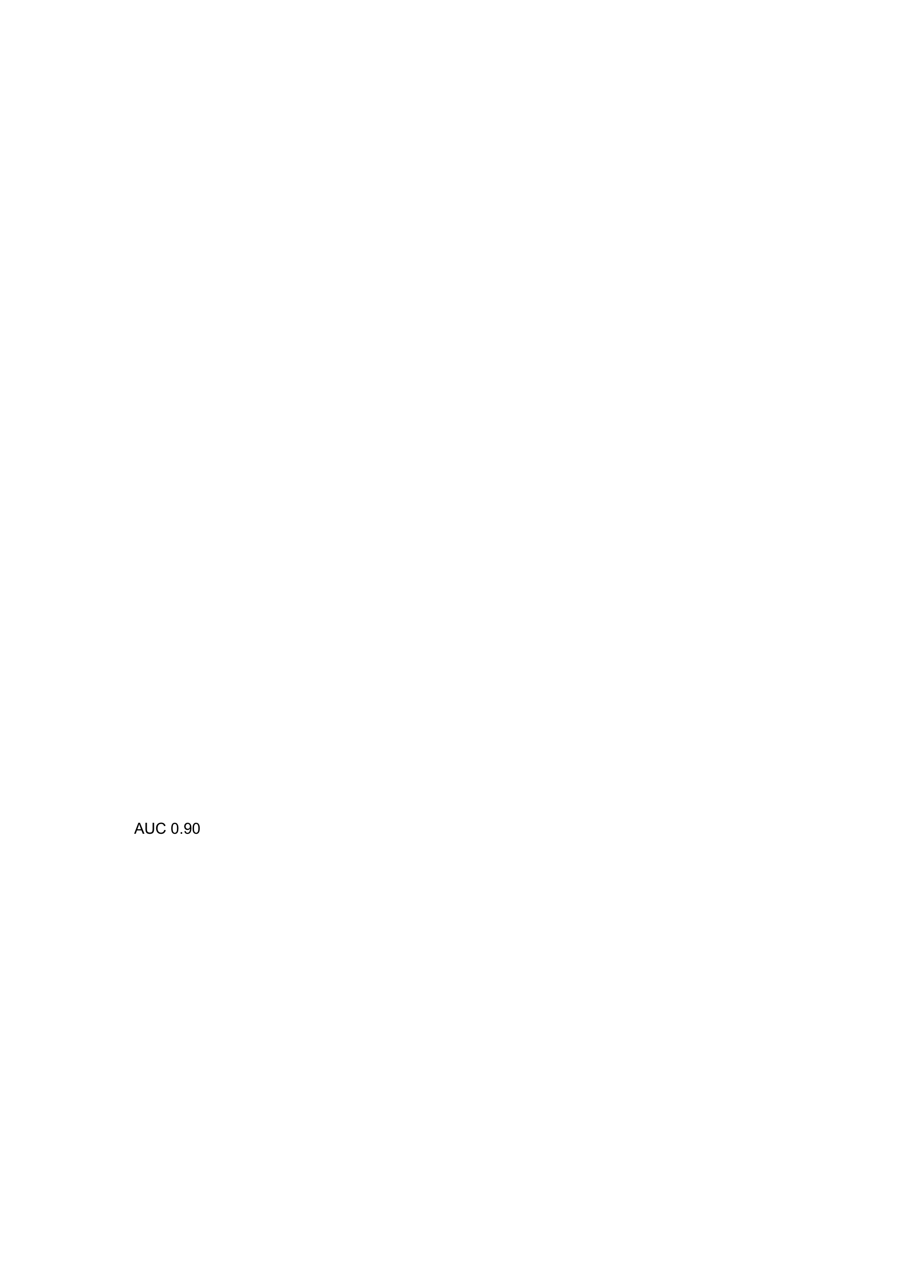


AUC 0.91

AUC 0.90

AUC 0.91

AUC 0.95

AUC 0.96

AUC 0.94

AUC 0.96

AUC 0.44

**Additional file 3: Figure S2 Area under the receiver operating characteristic (ROC) curve**

UC Rem(14) vs. Control

UC Rem(6) vs. Control

UC Rem(2) vs. Control

UC Rem(0) vs. Control

CD Rem(14) vs. Control

UC(0) vs. Control

CD(0) vs. Control

CD Rem(0) vs. Control

CD Rem(2) vs. Control

CD Rem(6) vs. Control
