# Supplementary material for: Metabonomics uncovers a reversible proatherogenic lipid profile during infliximab therapy of inflammatory bowel disease
Source: BMC Med. 2017 Oct 16;15:184. doi: 10.1186/s12916-017-0949-7 (PMC5641999; doi:10.1186/s12916-017-0949-7)
Supplement: Supplementary file 4 — Validation of PLS-DA and O-PLS-DA models. (DOCX 24 kb) [file 12916_2017_949_MOESM4_ESM.docx]

| **Model**  **Additional file 4: Table S2 Validation of PLS-DA and O-PLS-DA models** | **PLS-DA**  **Permutation test n=200**  Q^2^ | **O-PLS-DA**  **CV-ANOVA**  Q^2^ |
| --- | --- | --- |
| CD Res(0) vs. Control  CD Res(2) vs. Control  CD Res(6) vs. Control  CD Res(14) vs. Control | 0.692 ✓  0.632 ✓  0.598 ✓  0.568 ✓ | 0.637 ✓, p<0.001  0.586 ✓, p<0.001  0.574 ✓, p<0.001  0.557 ✓, p<0.001 |
| CD NRes(0) vs. Control  CD NRes (2) vs. Control  CD NRes (6) vs. Control  CD NRes (14) vs. Control | 0.706 ✓  0.339 ×  0.390 ✓  —— | 0.731 ✓, p<0.001  0.498 ✓, p<0.001  0.502 ✓, p<0.001  —— |
| CD Rem(0) vs. CD Res(0)  CD NRes (0) vs. CD Res(0)  CD NRes (0) vs. CD Rem(0) | 0.097 ×  0.628 ✓  0.195 × | 0.035 ×, p=0.89  0.558 ×, p=0.05  -0.143, × p=1.00 |
| UC Res(0) vs. Control  UC Res(2) vs. Control  UC Res(6) vs. Control  UC Res(14) vs. Control | 0.477 ✓  0.388 ✓  0.453 ✓  0.471 ✓ | 0.544 ✓, p<0.001  0.552 ✓, p<0.001  0.419 ✓, p<0.001  0.424 ✓, p<0.001 |
| UC NRes (0) vs. Control  UC NRes (2) vs. Control  UC NRes (6) vs. Control  UC NRes (14) vs. Control | 0.272 ×  0.597 ✓  0.155 ×  0.380 × | 0.313 ✓, p=0.003  0.584 ✓, p<0.001  0.052 ×, p=0.734  0.289 ✓, p=0.013 |
| UC Rem(0) vs. UC Res(0)  UC NRes (0) vs. UC Res(0)  UC NRes (0) vs. UC Rem(0) | -0.21 ×  -0.175 ×  -0.102 × | -0.296 ×, p=1  0.022 ×, p=1  -0.573 ×, p=1 |

The models were only considered valid if the permutation test and the CV-ANOVA test (p<0.05) were satisfied at the same time

CD, Crohn’s disease; CV-ANOVA, analysis of variance of the cross-validated residuals; NRes, non-responder, O-PLS-DA, orthogonal-projection to latent structure-discriminant analysis; PLS-DA, projection to latent structure-discriminant analysis; Rem, remission; Res, responder; UC, ulcerative colitis

(0), before 1^st^ infusion of infliximab; (2), before 2^nd^ infusion; (6), before 3^rd^ infusion; (14), before 4^th^ infusion

Q^2^, predictability of the model; r correlation coefficient

✓ valid model

X invalid model

-- Not enough samples to perform statistics
