# Supplementary material for: Metabonomics uncovers a reversible proatherogenic lipid profile during infliximab therapy of inflammatory bowel disease
Source: BMC Med. 2017 Oct 16;15:184. doi: 10.1186/s12916-017-0949-7 (PMC5641999; doi:10.1186/s12916-017-0949-7)
Supplement: Supplementary file 5 — Validation of PLS-DA and O-PLS-DA models of UC phenotypes and treatment response. (DOCX 23 kb) [file 12916_2017_949_MOESM5_ESM.docx]

| **Group**  **Additional file 5: Tables S3 Validation of PLS-DA and O-PLS-DA models of UC phenotypes and treatment response** | **Type** | **Model** | **PLS-DA**  **Permutation test, n=200**  Q^2^ | **O-PLS-DA**  **CV-ANOVA**  Q^2^ |
| --- | --- | --- | --- | --- |
| UC(0) | Gender | Female vs. Male | 0.258 | 0.215 |
| UC(0) | Age at sampling | ≤30 vs. 31-40 | -0.210 | -0.269 |
|  |  | ≤30 vs. ≥41 | -0.145 | -0.288 |
|  |  | 31-40 vs. ≥41 | 0.163 | -0.019 |
| UC(0) | Age at onset | ≤25 vs. >25 | 0.168 | 0.188 |
| UC(0) | Years with disease | ≤10 vs. >10 | -0.203 | -0.374 |
| UC(0) | Mayo score | Mild/moderate vs. Severe | 0.009 | 0.553 |
| UC(0) | Extension | Left-sided colitis^a^ vs. Pancolitis | 0.026 | 0.913 |
| UC(0) | Surgery | No surgery vs. Surgery | —— | —— |
| UC(0) | Smoking | Smoking vs. No smoking | —— | —— |
| UC(0) | EIM | Present vs. Not present | —— | —— |
| UC(0) | Steroid response | SD vs. SI; SD vs. SR, SI vs. SR | —— | —— |
| UC Rem | 0; 2; 6; 14 weeks | 0/2; 0/6; 0/14; 2/6; 2/14; 6/14 | × | × |
| UC Res | 0; 2; 6; 14 weeks | 0/2; 0/6; 0/14; 2/6; 2/14; 6/14 | × | × |
| UC NRes | 0; 2; 6; 14 weeks | 0/2; 0/6; 0/14; 2/6; 2/14; 6/14 | × | × |

The models were only considered valid if the permutation test and the CV-ANOVA test (p<0.05) were satisfied at the same time. None of the models in this table turned out to be valid

CV-ANOVA, analysis of variance of the cross-validated residuals; EIM, extra intestinal manifestations; NRes, non-responder; O-PLS-DA, orthogonal-projection to latent structure-discriminant analysis; PLS-DA, projection to latent structure-discriminant analysis; Rem, remission; Res, responder; SD, steroid dependence; SI, steroid independence; SR, steroid resistant; UC, ulcerative colitis

(0), before 1^st^ infusion of infliximab; (2), before 2^nd^ infusion; (6), before 3^rd^ infusion; (14), before 4^th^

infusion

Q^2^, predictability of the model;

X, invalid model;

- Not enough samples to make an analysis

^a^ Left-sided colitis includes patients with both proctitis, proctosigmoiditis, and complete left-sided colitis
