# Supplementary material for: Metabonomics uncovers a reversible proatherogenic lipid profile during infliximab therapy of inflammatory bowel disease
Source: BMC Med. 2017 Oct 16;15:184. doi: 10.1186/s12916-017-0949-7 (PMC5641999; doi:10.1186/s12916-017-0949-7)
Supplement: Supplementary file 6 — Validation of PLS-DA and O-PLS-DA models of CD phenotypes and treatment response. (DOCX 24 kb) [file 12916_2017_949_MOESM6_ESM.docx]

| **Group**  **Additional file 6: Tables S4 Validation of PLS-DA and O-PLS-DA models of CD phenotypes and treatment response** | **Type** | **Model** | **PLS-DA**  **Permutation test, n=200**  Q^2^ | **O-PLS-DA**  **CV-ANOVA**  Q^2^ |
| --- | --- | --- | --- | --- |
| CD(0) | Gender | Female vs. Male | -0.045 | -0.195 |
| CD(0) | Age | ≤30 vs. 31-40 | -0.013 | -0.656 |
|  |  | ≤30 vs. ≥41 | 0.029 | -0.205 |
|  |  | 31-40 vs. ≥41 | 0.333 | 0.073 |
| CD(0) | Age at onset | ≤25 vs. >25 | -0.030 | -0.260 |
| CD(0) | Years with disease | ≤10 vs. >10 | -0.210 | -0.227 |
| CD(0) | HB score | Mild vs. Moderate | 0.153 | 0.758 |
| CD(0) | Fistulizing disease | No vs. Yes | -0.117 | -0.017 |
| CD(0) | Stenosis | No vs. Yes | -0.136 | -0.265 |
| CD(0) | Surgery | No/surgery | 0.194 | -0.064 |
| CD(0) | Smoking | No smoking vs. smoking | 0.167 | 0.003 |
| CD(0) | EIM | Present vs. Not present | —— | —— |
| CD(0) | Steroid response | SD vs. SI | 0.327 | 0.250 |
|  |  | SD vs. SR | 0.239 | -0.376 |
|  |  | SI vs. SR | 0.138 | -0.020 |
| CD Rem | 0; 2; 6; 14 weeks | 0/2; 0/6; 0/14; 2/6; 2/14; 6/14 | × | × |
| CD Res | 0; 2; 6; 14 weeks | 0/2; 0/6; 0/14; 2/6; 2/14; 6/14 | × | × |
| CD NRes | 0; 2; 6; 14 weeks | 0/2; 0/6; 2/6 | × | × |

The models were only considered valid if the permutation test and the CV-ANOVA test (p<0.05) were satisfied at the same time. None of the models in this table turned out to be valid

CD, Crohn’s disease; CV-ANOVA, analysis of variance of the cross-validated residuals; EIM, extra intestinal manifestations; NRes, non-responder;O-PLS-DA, orthogonal-projection to latent structure-discriminant analysis; PLS-DA, projection to latent structure-discriminant analysis; Rem, remission; Res, responder; SD, steroid dependence; SI, steroid independence; SR, steroid resistant

(0), before 1^st^ infusion of infliximab; (2), before 2^nd^ infusion; (6), before 3^rd^ infusion; (14), before 4^th^ infusion

Q^2^, predictability of the model;

X, invalid model;

- Not enough samples to make an analysis

^a^ Due to heterogeneity in the affected section of the gastrointestinal tract no models for the extent could be created
