# Supplementary material for: Metabonomics uncovers a reversible proatherogenic lipid profile during infliximab therapy of inflammatory bowel disease
Source: BMC Med. 2017 Oct 16;15:184. doi: 10.1186/s12916-017-0949-7 (PMC5641999; doi:10.1186/s12916-017-0949-7)
Supplement: Supplementary file 7 — Correlation analysis between phenylalanine and tyrosine. (DOCX 49 kb) [file 12916_2017_949_MOESM7_ESM.docx]

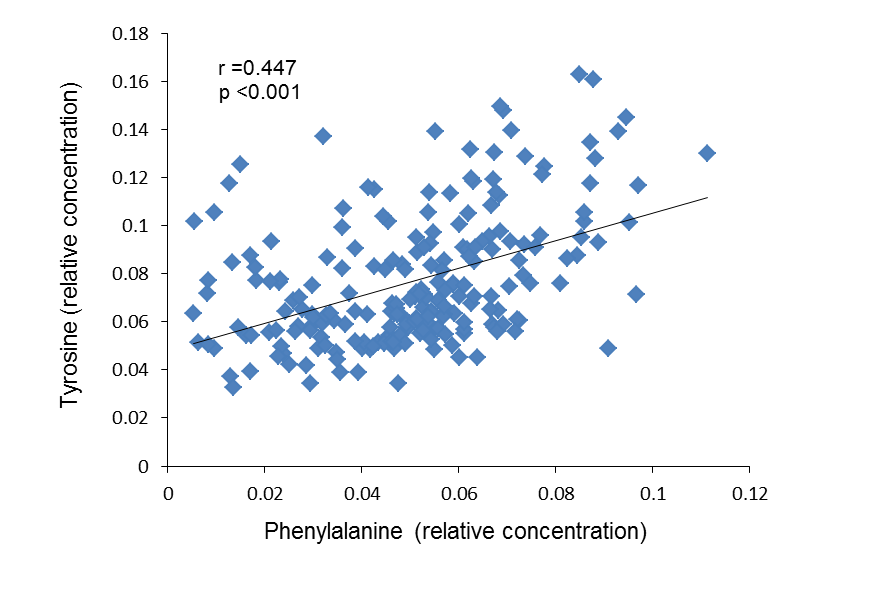


Correlation analysis between phenylalanine and tyrosine according to the relative concentration (integral area) from both CD and UC patients (CD(0) and UC(0)).

**Additional file 7: Figure S3 Correlation analysis between phenylalanine and tyrosine**
